# Inactivation of a Single Copy of *Crebbp* Selectively Alters Pre-mRNA Processing in Mouse Hematopoietic Stem Cells

**DOI:** 10.1371/journal.pone.0024153

**Published:** 2011-08-25

**Authors:** Madeleine E. Lemieux, Ziming Cheng, Qing Zhou, Ruth White, John Cornell, Andrew L. Kung, Vivienne I. Rebel

**Affiliations:** 1 Department of Pediatric Oncology, Dana-Farber Cancer Institute and Harvard Medical School, Boston, Massachusetts, United States of America; 2 Greehey Children's Cancer Research Institute (GCCRI), The University of Texas Health Science Center at San Antonio (UTHSCSA), San Antonio, Texas, United States of America; 3 Department of Cell and Developmental Biology, Oregon Health and Science University, Portland, Oregon, United States of America; 4 Department of Epidemiology and Biostatistics, The University of Texas Health Science Center at San Antonio (UTHSCSA), San Antonio, Texas, United States of America; 5 Department of Cellular and Structural Biology, The University of Texas Health Science Center at San Antonio (UTHSCSA), San Antonio, Texas, United States of America; Emory University, United States of America

## Abstract

Global expression analysis of fetal liver hematopoietic stem cells (FL HSCs) revealed the presence of unspliced pre-mRNA for a number of genes in normal FL HSCs. In a subset of these genes, *Crebbp*+/**−** FL HSCs had less unprocessed pre-mRNA without a corresponding reduction in total mRNA levels. Among the genes thus identified were the key regulators of HSC function *Itga4*, *Msi2* and *Tcf4*. A similar but much weaker effect was apparent in *Ep300*+/**−** FL HSCs, indicating that, in this context as in others, the two paralogs are not interchangeable. As a group, the down-regulated intronic probe sets could discriminate adult HSCs from more mature cell types, suggesting that the underlying mechanism is regulated with differentiation stage and is active in both fetal and adult hematopoiesis. Consistent with increased myelopoiesis in *Crebbp* hemizygous mice, targeted reduction of CREBBP abundance by shRNA in the multipotent EML cell line triggered spontaneous myeloid differentiation in the absence of the normally required inductive signals. In addition, differences in protein levels between phenotypically distinct EML subpopulations were better predicted by taking into account not only the total mRNA signal but also the amount of unspliced message present. CREBBP thus appears to selectively influence the timing and degree of pre-mRNA processing of genes essential for HSC regulation and thereby has the potential to alter subsequent cell fate decisions in HSCs.

## Introduction

Cyclic-AMP-responsive element binding protein (CREB) binding protein (CREBBP) – more commonly referred to as CBP – is a multifunctional protein which facilitates gene expression through several mechanisms, including chromatin remodeling, acetylation of associated proteins, and recruitment of the basal transcription machinery to promoters [1]. We have previously shown that CREBBP and its paralog EP300 are essential for proper hematopoietic stem cell regulation but are nevertheless not functionally redundant in this setting: both copies of *Crebbp* are essential for HSC self-renewal while *Ep300*+/**−** HSCs are not compromised in this respect[2]. Inactivation of a single copy of the *Crebbp* gene results in multi-lineage defects in differentiation with a clear excess in myeloid cell production and an age-dependent increase in the incidence of hematologic malignancies[3].

CREBBP also acts as a scaffold in numerous protein-protein interactions [4] so that changes in its levels have the potential to broadly affect cellular processes by altering multiple signaling and transcriptional pathways. In particular, it has the potential to act as a signal integrator within the hematopoietic system[5] through its interaction with both ubiquitous transcription factors such as SP1[6], [7] and the glucocorticoid receptor (NR3C1) [8], [9] and with factors like SFPI1/PU.1[10] and C/EBPalpha[11] which are essential for HSC function[12], [13].

In addition to its activities as coactivator and integrator, confocal microscopy studies have localized CREBBP to nuclear speckles containing splicing proteins[14], [15] and it has been shown to regulate 3′-end processing[16]. Both CREBBP and EP300 have furthermore been shown to be concentrated at both 5′ and 3′ ends of genes with which they associate[17]. It thus appears that CREBBP is involved in pre-mRNA maturation. In addition, experiments in macrophages[18] and T-cells[19] have shown CREBBP/EP300 to be present at the promoters of early response genes, even in the absence of stimulus, and associated with the production of full-length, unspliced transcripts. Other recent studies have reported that a large majority of genes with a paused polymerase produce full-length transcripts, although often at levels below detection by expression microarrays[20] and RNA-seq studies have documented the presence of low-abundance intronic sequences in B-cell, kidney[21] and embryonic stem cell lines[22]. It has also been noted that HSCs prime multiple lineage programs prior to commitment decisions[23] and that HSCs normally contain unspliced transcripts that disappear as HSCs are driven to proliferate and differentiate[24]. Destabilization of this primed state has been proposed as a first stage of a cascade towards differentiation[25]. The general model that emerges from these findings is that unspliced, full-length transcripts are produced as a means of bookmarking loci and keeping them in an open chromatin state to facilitate subsequent rapid transcriptional up-regulation[18], [19].

The presence of unspliced transcripts in HSCs and the links between CREBBP and EP300 with the constitutive production of unspliced RNA and with pre-mRNA processing prompted us to examine more closely an anomaly we had noted in microarray-based gene expression studies but had previously attributed to experimental “noise”. We had noticed that more than half of the probe sets down-regulated in *Crebbp*+/**−** FL HSCs relative to wild-type (WT) mapped entirely within introns, rather than detecting exonic or spliced sequences. We therefore set out to test whether this might be evidence that reduced CREBBP levels selectively alter the generation of full-length, unspliced pre-mRNA. We also asked whether this process might be associated with differentiation since self-renewal and lineage commitment are both responses for which HSCs are primed.

As predicted by our microarray studies, we found that several genes associated with HSC function showed variable ratios of intronic to total RNA signal in *Crebbp*+/**−** FL HSCs relative to WT by quantitative RT-PCR (qRT). In addition to primary FL HSCs, we looked for CREBBP-associated changes in mRNA, intronic message and protein levels in multipotent EML cells which retain lymphoid, myeloid and erythroid potential in the presence of Stem Cell Factor (SCF) but can be induced to undergo differentiation by treatment with appropriate stimuli[26]. Targeted reduction of *Crebbp* by shRNA in EML cells was sufficient to trigger widespread myeloid differentiation of EML cells, bypassing their usual requirement for withdrawal of SCF and treatment with retinoic acid, interleukin-3 (IL-3) and granulocyte-macrophage colony stimulating factor (GM-CSF). A subset of genes tested also showed altered levels of intronic message in subpopulations of EML cells at different phenotypically-defined stages of development which corresponded to changes in protein abundance not predicted by full-length mRNA levels. Furthermore, the differences in intronic levels correlated with differentiation stage-dependent changes in CREBBP levels. Taken together, our data suggest a novel, cell type-specific function for CREBBP in regulating the timing and extent of pre-mRNA splicing of key regulators of HSC maintenance and function.

## Results

### Down-regulation of intronic probe sets without proportional changes in total mRNA levels

We have carried out a global analysis of expression profiles of WT and *Crebbp*+/**−** FL HSCs. Our in-house annotation of the Affymetrix Mouse 430 2.0 microarrays (see Materials and Methods for details) indicated that ∼60% of down-regulated probe sets - but none of the up-regulated ones - mapped on the coding strand but entirely within intronic regions of genes (Table 1 and Table S1). This is a significant enrichment over the array background of 15% (6780/45037, excluding Affymetrix control probe sets, χ^2^ test p <1.1×10^−15^). Of the 83 intronic probe sets representing 77 distinct transcripts (both protein-coding and non-coding genes), 13 were designed to detect expressed sequence tags (ESTs) in libraries constructed from adult HSCs[24], [27], [28]. In total, there was evidence in the EST database for intronic message for 50/77 transcripts (Table S1). We will refer to these probe sets and their associated genes as “intronic probe sets” and “intronic targets”, respectively, to distinguish them from “mRNA probe sets” and “exonic probe sets” detecting spliced mRNA or 3′-most exons. Notably, of the 74/77 transcripts with both intronic and mRNA probe sets, we found changes in total mRNA levels for only 2: *Mbnl1* and *Meis1*. In both cases, the mRNA probe set could detect either a putative (non-RefSeq) alternative 3′ exon or an intron in a longer isoform.

**Table 1:** Distribution of Probe Sets Differentially Expressed Relative to Matched Wild-type Cells.

	*Crebbp*+/− HSC		*Ep300*+/− HSC		*Cdkn1a*−/− HSC		*Crebbp*+/− MEF	
Probe set target	Up	Down	Up	Down	Up	Down	Up	Down
**mRNA**								
RefSeq	48	27	4	12	5	48	18	33
non-RefSeq*	6	0	1	2	0	1	0	5
ncRNA	0	3	0	0	0	1	0	1
putative ncRNA	1	2	0	0	2	4	0	0
putative alt 3′UTR	5	14	0	1	0	3	0	0
**Total mRNA**	60	46	5	15	7	57	18	39
**intronic (expected^†^)**	**0 (15)**	**83 (15)**	**0 (3)**	**15 (3)**	**3 (7)**	**15 (7)**	**0 (5)**	**1 (5)**
repeat	0	6	0	0	0	3	0	0
ambiguous/unclear	1	1	0	0	0	6	0	2

An attenuated bias was found in an *Ep300*+/**−** FL HSC expression data set we produced concurrently in which 15/35 differentially expressed probe set were intronic (χ^2^ test p = 1.3×10^−5^), again all of them down-regulated. In contrast, probe sets altered in *Crebbp*+/**−** mouse embryonic fibroblasts (MEFs) relative to WT MEFs showed no enrichment for intronic probe sets (Table 1), indicating that the effect is cell type-specific. Like CREBBP, CDKN1A (p21^Cip1/Waf1^) is required for HSC self-renewal and its loss leads to exhaustion of the HSC pool upon serial transplantation[29]. Unlike *Crebbp*+/**−** FL HSCs, however, FL HSCs null for *Cdkn1a* showed both up- and down-regulated intronic probe sets at a frequency close to predicted relative to their WT controls (Table 1). The change in intronic sequence abundance is thus not common to all FL HSCs with compromised self-renewal ability.

### Down-regulated intronic probe sets as a discriminating HSC signature

To determine whether these differentially expressed intronic probe sets were functionally relevant, we first asked whether the intronic probe sets down-regulated in *Crebbp*+/**−** FL HSC could distinguish HSCs from more mature cell types by hierarchical clustering. We took advantage of previously published data sets available for download from the Gene Expression Omnibus (http://www.ncbi.nlm.nih.gov/geo/). In the absence of appropriate data sets from fetal liver, we selected expression series comparing adult bone marrow (BM) HSCs and various populations of early progenitors and more mature hematopoietic cells[30], [31], [32]. The fetal liver is the primary site of hematopoiesis from day 11 of mouse development, a function taken over by the bone marrow after birth[33]. Although FL HSCs are phenotypically and functionally distinguishable from BM HSCs, FL HSCs are capable of fully reconstituting long-term hematopoiesis in adult animals[34], [35] and unspliced transcripts had previously been detected in adult BM[24].

**Figure 1:**
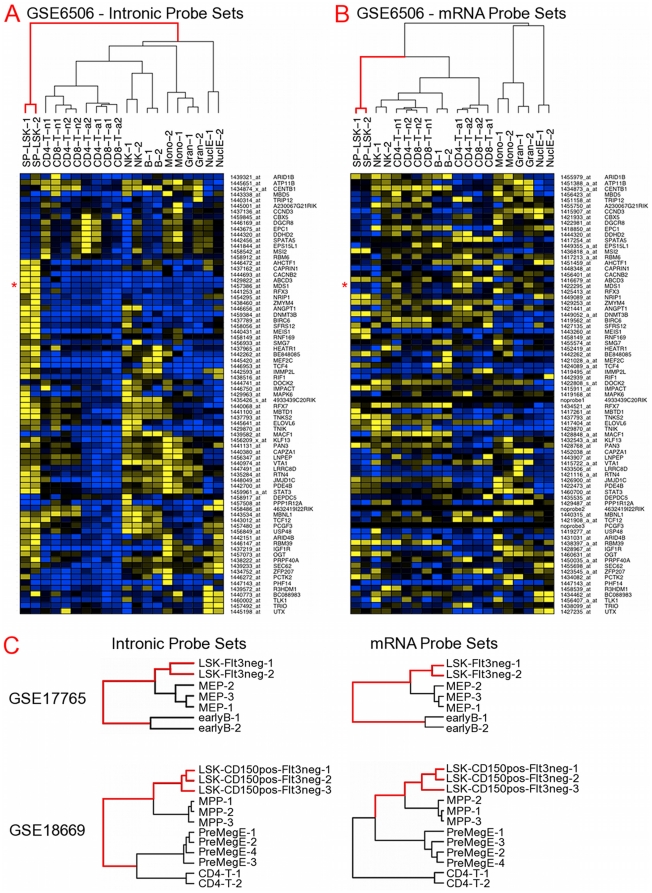
Discriminatory power of intronic probe sets in hierarchical clustering of hematopoietic cell populations. (A) Hierarchical clustering of expression data from Chambers *et al* [31] using intronic probe sets down-regulated in *Crebbp*+/**−** vs WT HSCs. Cell types represented are adult HSCs (SP-LSK, red branch on the dendrogram), activated (a) and naive (n) CD4+ and CD8+ lymphocytes, natural killer (NK) cells, B cells, nucleated erythrocytes (NuclE), monocytes (Mono), and granulocytes (Gran). (B) Same data set as in (A) but with cell populations clustered using mRNA probe sets for the same target genes. In cases where multiple probe sets were available, the one with the largest difference in *Crebbp*+/**−** vs WT HSCs was used. Genes are shown in the same order as in (A) with HSC branches shown in red to the nearest junction point with a mature cell type subtree. (C) Clustering as in (A) (left) and (B) (right) of 2 other published data sets[30], [32] with different subpopulations. As before, red dendrogram branches highlight the distance from HSCs to the nearest mature cell type junction point. LSK: Lin- SCA1+ KIT+; SP: side population; LK: Lin- KIT+; MEP: megakaryocyte/erythrocyte progenitor, LK SCA1- CD34- Fc-gammaRII/III-; MPP: multi-potential progenitor, LSK CD150- FLT3+; PreMegE: megakaryocyte/erythrocyte progenitors, LK SCA1- CD150+ CD105- CD41-.

We used either all 83 probe sets for clustering or the single most highly expressed probe set per transcript (77 in total) to avoid overweighting genes detected by multiple intronic probe sets. The cell populations clustered the same way with both approaches. Figure 1A shows the heat map and sample dendrogram for GSE6506[31]. The two adult BM HSC (SP-LSK) samples of this data set are grouped together on a branch (indicated in red) distinct from all the more differentiated cell types. As a control, we generated 10,000 random samples of 83 probe sets annotated as being both intronic and expressed in WT or *Crebbp*+/**−** FL HSCs. Only 0.52% of the random samples yielded such a clean distinction of HSCs from mature cell types, arguing against the separation occurring purely by chance and suggesting that the underlying process may be common to both adult and fetal HSCs.

Are the intronic probe sets simply surrogate measures of the total mRNA level? For each gene with at least one mRNA probe set (74/77 genes), the one with the largest difference in the *Crebbp*+/**−** vs WT FL HSC data set was retained. Figure 1B shows the heat map for the same genes as 1A and in the same row order but with the various cell populations clustered by mRNA probe set. In this case, the HSC branch (shown in red) is no longer clearly distinct from mature cell types but is part of a subtree including all lymphoid cell types. Since the two heat maps are row-normalized, it is clear that the relative expression levels of intronic and mRNA probe sets for the same gene are often quite different (for example, compare the relative levels for *Mds1*, marked by a red asterisk in both Figure 1A and 1B). This is inconsistent with intronic transcript levels being a constant proportion of total message produced in all cell types.

Hierarchical clustering of 2 other published data sets were carried out in the same manner (Figure 1C). In each case, the intronic probe sets (Figure 1C left) segregated the experiment's HSCs (indicated by red bars) from mature cell types. In addition, hierarchical clustering using mRNA probe sets (Figure 1B and 1C right) resulted in somewhat different clustering tree structures than those generated using intronic probe sets (Figure 1A, 1C left), depending on the populations involved. Where intronic and mRNA clustering dendrograms are noticeably different (based on total distance along the dendrogram arms and positions of branch points), the intronic probe sets separate HSCs from early progenitors better than do the mRNA probe sets (shorter overall distance, different branches). The changes in intron levels are therefore not strictly reflections of those in total mRNA differences.

**Figure 2:**
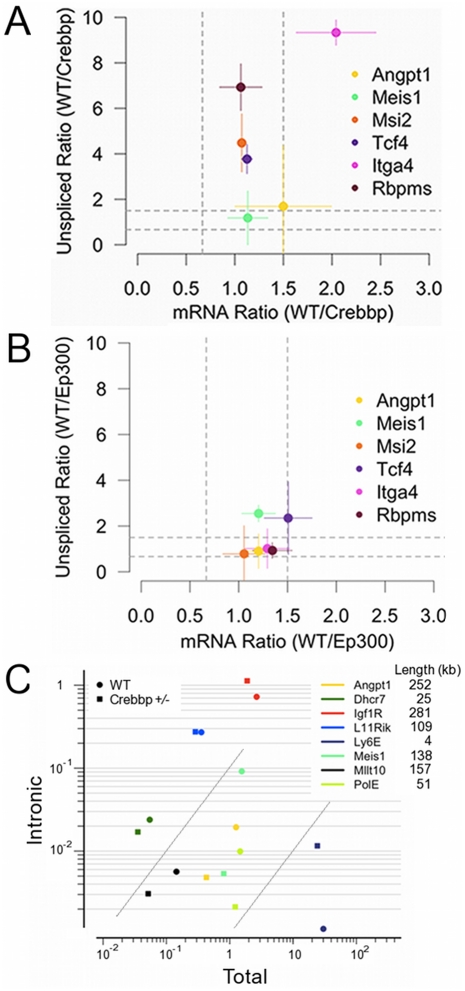
Changes in the ratio of unspliced message relative to total mRNA ratios in WT *vs Crebbp*+/− HSCs. (A) WT/*Crebbp*+/**−** ratios for total mRNA *vs* unspliced mRNA qRT signal for the indicated genes. Dashed grey lines mark the 1.5-fold limit used as a cut-off for differential expression. Points lying within the vertical grey lines but above the horizontal ones indicate little change in mRNA levels in WT *vs Crebbp*+/**−** cells but a reduction in the ratio of unspliced message. Plotted are median values with horizontal and vertical lines extending to indicate median absolute deviation for the mRNA and unspliced ratios, respectively. (B) WT/*Ep300*+/**−** ratios for total mRNA *vs* unspliced mRNA qRT signal for the indicated genes as in (A) showing little if any discrepant change in total and unspliced transcript levels. (C) Total mRNA vs Intronic qRT signal for the indicated genes in WT and *Crebbp*+/**−** HSCs. Grey lines indicate a slope of 1 on the log-log plot and are provided as a visual reference only. Gene lengths in kilobases (kb) are shown at the right.

Encouraged by this evidence that the presence of unspliced message was non-random and the finding that our intronic signature was associated with HSC differentiation, we then carried out 3 independent qRT experiments in purified FL HSCs with primers designed to detect spliced or unspliced transcripts (Table 2). Follow-up targets were selected based on previous reports of their involvement in HSC function[36], [37], [38], [39], [40], [41] or, in the case of *Rbpms*, for its implication in developmental processes[42] and physical association with SMAD4[43], a factor critical for HSC self-renewal[44]. For exonic amplicons, results from qRT consistently agreed well with the microarray data (Table S2), showing little if any change between WT and *Crebbp*+/**−** samples. For intronic amplicons, the reproducibility of the results depended on the proximity of the amplicon to poly(A) tracts of sufficient length to potentially prime the oligo(dT) reverse transcription[45] that we used to amplify our starting material (Table S2). Despite this technical limitation, we found that *Tcf4/E2-2* and *Msi2* intronic amplicons both showed reduced levels in *Crebbp*+/**−** HSC relative to WT. Two other genes, *Itga4* and *Rbpms*, which had down-regulated intronic probe sets (although only to a p-value <0.1 significance level in the microarray data) also exhibited greater reduction in intronic than total signal. Figure 2 shows a summary of these results. Plotted are the ratios of total mRNA and intronic signal of WT FL HSCs relative to either *Crebbp*+/**−** (A) or *Ep300*+/**−** (B). Genes for which the ratio is similar for total mRNA and unspliced transcripts fall between the vertical and horizontal grey lines marking 1.5-fold differences. *Tcf4*, *Msi2* and *Rbpms*, on the other hand, fall within the vertical lines, indicating similar mRNA levels in WT and *Crebbp*+/**−** HSCs, but lie well above the horizontal line marking a greater than 1.5-fold excess intronic signal in WT cells. *Itga4* mRNA levels decreased roughly 2-fold in *Crebbp*+/**−** HSCs relative to WT but unspliced transcript levels are reduced even more substantially (∼10-fold). Consistent with the array data, *Ep300*+/**−** samples showed attenuated, if any, difference in intronic and total mRNA ratios (Figure 2B).

**Table 2:** Primer Sequences Used in qRT Reactions.

Gene	Amplicon Type	Forward (5′->3′)	Reverse (5′->3′)
*Crebbp*	exonic	AAGTCACCCAGCTCTCCTCA	GGCTGATTGGCCACATACTT
*Ep300*	exonic	GCCTTCTCCACACCATGTTT	CGAGCTGTGAAAGCATTGAA
*Gapdh*	exonic	ACGTGCCGCCTGGAGAA	CATGCCTGCTTCACCACCTT
*Ly6E*	intron-spanning	CTTCCAACATGAGAGTCTTCC	GCAGATAACGTGATACAGTAATGG
	exon-intron	GTTGTCATTGGCTGGCTTTT	GCAGATAACGTGATACAGTAATGG
*1700081L11Rik*	intron-spanning	AAGCGTTTGATTCGGATGTC	CTGTTGTATGATCTGGGAAAGG
	exon-intron	AAGGGAAAGGCCTCTGGTTA	CTGTTGTATGATCTGGGAAAGG
*Dhcr7*	intron-spanning	GATTGTAGCCTGGACCCTCA	GAGAGCTGCACAGGGTGGTA
	exon-intron	GAAGCTGGCGTGACAAGTCT	GAGAGCTGCACAGGGTGGTA
*Pole*	intron-spanning	CTGATGGCCTTCACACTTCA	CTTTTGGAGCAGGCAGTAGG
	exon-intron	CTGATGGCCTTCACACTTCA	TGCTCTGCTCCTGCACTCTA
*Mllt10*	intron-spanning	TTCCATGCAGTATCGACATGA	TGACCAGATGACTGCTGAGG
	exon-intron	AGGGAAGGGATCAAGGAGAA	TGACCAGATGACTGCTGAGG
*Igf1r*	intron-spanning	AGCCCATGTGTGAGAAGACC	ACGCAGGTTGTGTTGTCGT
	exon-intron	AGCCCATGTGTGAGAAGACC	CGGAGCAGAAAGTGGAAGTC
	intronic	GTTGTGCCCCAGTGTTTTCT	TGGTCCTCAGCCAAGAGACT
*Angpt1*	intron-spanning	TCTGCCAGCTGTGTCTTGTT	CGTGTGGTTTTGAACAGCAT
	exon-intron	TCTGCCAGCTGTGTCTTGTT	CGTGTGGTTTTGAACAGCAT
	exonic	GCGCTGGCAGTACAATGACAGTTT	CACATTGCCCATGTTGAATCCGGT
	intronic	ACAACAGAGGCCACAAGCCTTAGA	CACCAGCCACTGCACAAAGACATT
*Meis1*	intron-spanning	ACAATTTCTGCCACCGGTAT	GTTCCTCCTGAACGAGTGGA
	exon-intron	CCCTGTAGCCCTCCTCAGTT	GTTCCTCCTGAACGAGTGGA
	exonic	TAGGAGGAGGCATGGAAACCCAAA	AGGTGATGGTTGTTGTTGCATGGG
	intronic	TGTGGTCCTATTTGCCCACTGCTA	TGCAACTCCATCCTCTGACACCTT
*Tcf4*	exonic	AGGACGAAATGCTGACCCTGAAGT	CGGGCAATGCTGCATGTAATTCCT
	intronic	AACTCCCGTGGGAATGAGTGATGT	ATGCCCACTGCCAACATCATGAAC
*Msi2*	exonic	TGGTACTTAAGGCACAGCCAGTGT	TTCTGCGCTATCCCGTCTGTGAAT
	intronic	TGCAGAAGCCAGCAAGTAGACTGT	AGGACTCGCTAGCCAAGATGGTTT
*Rbpms*	exonic	GGCAAACACGAAGATGGCCAAGAA	TATGGCTCCCTGGCAATGAACTGA
	intronic	AGGTTCCTGCCAATCCTGTCCTTT	AAACTGTGCTGCAAGGGTTCCAAG
*Itga4*	exonic	AGACCTTTGTACCTTTGCCTCCCA	AGAGAGAGCCTTCCCTGTTTGCTT
	intronic	GCAGGACATTCAAGTTGCCCTTGT	AGGAATTCCCACCTGCTACCAACA

Many of our target genes were >100 kilobases long and their mRNA was detected at modest levels on the microarrays. Consequently, we wondered whether changes in intronic sequence detection was a widespread phenomenon and whether it correlated with transcript length or expression level. We therefore carried out a further set of qRT reactions using linked primer pairs for which either the forward or the reverse primer was common to both the exonic and intronic amplicons for a given gene (see Table 2 for details). We included genes of varying lengths and predicted expression levels based on the microarray data. Figure 2C shows total and intronic mRNA levels for each gene in WT and *Crebbp*+/**−** HSCs. As can be seen in the figure, not all transcripts were altered in *Crebbp*+/**−** HSCs (e.g. *170081L11Rik* indicated as “L11Rik”). In some cases, both intronic and total signal changed proportionately (e.g. *Dhcr7*, *Mllt10* where the slope from the WT square to the *Crebbp*+/**−** circle is close to 1) while in others the variation was greater in the intronic signal than in total mRNA level (e.g. *Ly6*, *Pole, Meis1*). The change in intronic signal relative to total message thus seems gene-specific rather than a function of length or mRNA abundance (compare *Ly6E*, *Igf1R* and *Angpt1*, for example). Overall, the clustering and qRT results indicate that the mechanism underlying the alterations in intronic signal is both cell-type specific and selective in its targets.

### Classification of genes hosting differentially expressed intronic probe sets

We found no particular gene ontology term or pathway significantly over-represented among the 77 distinct transcripts hosting these 83 intronic probe sets. The list of functional categories represented by their encoded proteins, however, included kinases as well as genes associated with transcription, cell cycle regulation and cellular division, growth regulation and organismal development, chromatin binding and modification, and RNA binding and processing (Table 3). This last category is notable as it suggests that CREBBP might indirectly modulate intronic signal levels by regulating the expression of proteins that are themselves involved at different stages of RNA metabolism. Furthermore, CREBBP itself has also been localized by immunofluorescence to splicing speckles[14], [15] suggesting that it may also play a direct role in the splicing process. Interestingly, among the non-intronic targets summarized in Table 1 and listed in Table S1 are 2 down-regulated probe sets that detect the non-coding RNA, *Malat1*, which is also associated with splicing speckles[46] and regulates alternative splicing of pre-mRNA[47]. CREBBP may thus participate at several levels, both directly and indirectly, in a feed-forward loop regulating pre-mRNA processing.

**Table 3:** Functional Annotation of Intronic Target Genes.

**Cell cycle & division**	AHCTF1	**Kinase activity**	CENTB1
	CCND3		IGF1R
	MACF1		MAPK6
	MAPK6		PCTK2
	RIF1		TLK1
	TLK1		TNIK
			TRIO
**Chromatin binding**	ARID4B	**RNA binding &**	AHCTF1
**& modification**	CBX5	**processing**	DGCR8
	DNMT3B		MBNL1
	EPC1		MSI2
	JMJD1C		PAN3
	MBDT1		PRPF40A
	TLK1		RBM39
	UTX		SFRS12
			SMG7
**Development & growth**	AHCTF1		
	ANGPT1		
	MBNL1		
	MEF2C		
	MEIS1		
	RTN4		
	SPATA5		
	TCF4		
	TCF12		

We do not see a generalized impact on intronic levels with the reduction in *Crebbp* expression, however, indicating that there is some selectivity in the genes exhibiting these changes. We found evidence that the promoters of genes with differentially expressed intronic probe sets are enriched for a different set of transcription factor binding sites (TFBS) proximal to their transcription start sites (TSS, -500bp to +250bp) relative to the exonic target genes (Table 4 and Table S1). TFBS for ARID3A/BRIGHT, C/EBPalpha, GFI1, IRF1, NR3C1 (glucocorticoid receptor), SOX5 or SOX17 where significantly enriched only in the intronic target set. At least one of these TFBS was present in each of the intronic target promoters and 81% of promoters had sites for 3 or more of these transcription factors, all of which are expressed in FL HSCs based on our microarray data. Each of these factors except SOX5 has been implicated in hematopoietic regulation at various stages[13], [48], [49], [50], [51], [52] and CREBBP has been shown to interact (directly or by homology with EP300) with C/EBPalpha, IRF1, NR3C1 and multiple SOX family members[4].

**Table 4:** Binding Sites for Expressed Transcription Factors Enriched in Proximal Promoters of Target Genes.

			Intronic (all down)			Down mRNA			Up mRNA		
			N = 85, 61% (G+C)			n = 48, 59% (G+C)			n = 55, 60% (G+C)		
				EnrichmentP-value			EnrichmentP-value			EnrichmentP-value	
JasparTFBS Matrix	Factor	Binds CREBBP? †	Score *	TSS	Chr19	Score	TSS	Chr19	Score	TSS	Chr19
MA0079.2	SP1	Y	110	0.005	0						
MA0080.2	SFPI1/PU.1	Y	56.4	0	0				37.7	0	0
MA0152.1	NFATC2	Y	44.5	0.008	0.002				35.1	0	0
MA0098.1	ETS1	Y	25.9	0	0				19.6	0	0
MA0050.1	IRF1	Y	20.2	0	0						
MA0113.1	NR3C1/GR	Y	20.2	0	0						
GFI1_Q6 #	GFI1		11.2	0	0						
MA0151.1	ARID3A		8.88	0	0						
MA0028.1	ELK1	Y	8.82	0	0	2.42	0.006	0.001	13.6	0	0
MA0062.2	GABPA	Y	6.81	0	0				15.7	0	0
MA0078.1	SOX17		6.39	0	0.001						
MA0108.2	TBP	Y	5.63	0.002	0						
MA0087.1	SOX5		5.61	0.001	0						
MA0102.2	CEBPA	Y	4.73	0.001	0						
MA0060.1	NFYA	Y				10.5	0.001	0	6.4	0.004	0
MA0018.2	CREB1	Y				1.93	0.001	0			

† Bedford et al. (2010) and references therein.

*Scores comparable within but not between groups.

#From Transfac; Matys et al. (2003).

Taken together, these results indicate that reduction in CREBBP level in HSCs alters pre-mRNA processing in a regulated manner for a subset of genes. It is difficult to obtain sufficient material reproducibly from FL or adult HSC for in-depth molecular analyses so we turned for our subsequent studies to HSC-like EML cells which possess lymphomyeloid differentiation potential[26] and have previously been used as a model system for studying hematopoietic lineage commitment[53], [54], [55].

### Differentiation-associated down-regulation of CREBBP and spontaneous commitment to myeloid lineages upon knock-down of *Crebbp* expression in EML cells

**Figure 3:**
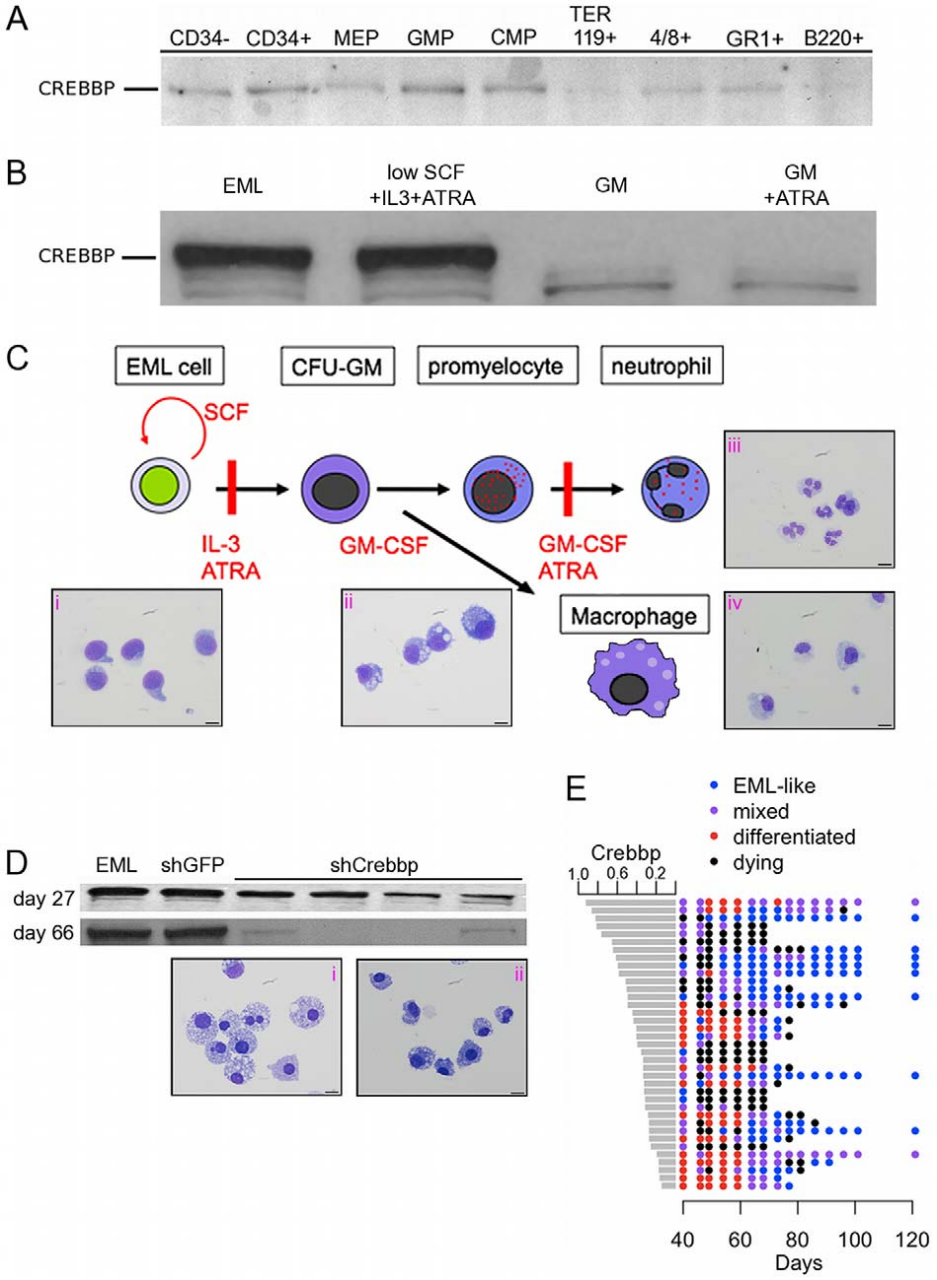
Knock-down of CREBBP levels triggers stimulus-independent myeloid differentiation of EML cells. (A) Western blots for CREBBP in lysates from 15,000 sorted hematopoietic cells per lane for a single experiment. Sort phenotypes: CD34- = LT HSCs (Lin- SCA1+ KIT++ CD34-), CD34+ = ST-HSC (Lin- SCA1+ KIT++ CD34+), MEP =  (Lin- SCA1- KIT+ CD34- CD16/32-), GMP =  (Lin- SCA1- KIT+ CD34+ CD16/32+), CMP =  (Lin- SCA1- KIT+ CD34+ CD16/32-), others as indicated. (B) Western blots for CREBBP in 12.5 µg of nuclear extracts of undifferentiated EML cells (EML) and EML cells induced to differentiate by reducing the dose of SCF and addition of IL3 and ATRA (low SCF+IL3+ATRA), switching to GM-CSF only (GM) and then adding ATRA (GM+ATRA). Shown is a representative of at least 3 experiments. (C) Depiction of factors required to either maintain undifferentiated EML cell cultures (SCF) or to trigger myeloid differentiation (IL-3, ATRA, GM-CSF). Vertical red lines indicate RARα403 blocks to differentiation and the factors used to overcome them. (i-iv) Representative cytospins stained with Wright-Giemsa. (i) Undifferentiated EML cells with characteristic hand-mirror morphology. (ii) EML cells in low SCF + IL-3 + ATRA. (iii, iv) Fully differentiated EML cells after culture in GM-CSF alone followed by GM-CSF + ATRA. (D) CREBBP protein levels in 3×10^5^ cloned cells, either control (EML, shGFP) or in representative clones expressing shRNA against *Crebbp* measured at the indicated times post-cloning. (i, ii) Representative cytospins of cultures treated with shCrebbp. Scale bar in cytospins  = 10 µm at 60X magnification. (E) Summary of outcomes for clones expressing shRNA against *Crebbp*. Each point represents the state of the culture at the indicated time. Relative *Crebbp* levels measured at 5 weeks post-cloning are indicated by the histogram on the left. Growth characteristics are indicated by color: EML-like (rapid growth with <5% adherent cells), mixed (<50% adherent), differentiated (>50% adherent), dying (>50% cells dead). Clone histories were recorded for 120 days or ended when the clone could no longer be passaged.

*Crebbp* expression is widespread but developmentally regulated during embryogenesis[56] and its transcript[57] and protein levels (Figure 3A) are lower in certain committed progenitors and mature cells than in HSCs. Similarly, EML cells driven to myeloid differentiation by growth factor and all-trans retinoic acid (ATRA) stimulation down-regulate CREBBP levels (Figure 3B). Parental EML cells (Figure 3C i) showed a characteristic hand-mirror morphology that was lost as the cells began differentiating upon reduction of SCF and addition of IL-3 and ATRA (Figure 3C ii). As previously reported[53], the shift to medium containing only GM-CSF resulted in some cell death but cultures further stimulated with GM-CSF + ATRA gave rise to mature granulocytes (∼70% of cells, Figure 3C iii) mixed with macrophages (Figure 3C iv).

Cells exposed to control shRNA against GFP continued to express CREBBP at WT levels (compare Figure 3D EML and shGFP) and grew in suspension like parental EML cells. In contrast, reduction of CREBBP levels by lentiviral delivery of shRNA led to widespread death or stimulus-independent differentiation of EML cells (Figure 3D i–ii).

Forty *Crebbp* knock-down (shCrebbp) clones were isolated in SCF-containing methylcellulose and then passaged in liquid culture in the presence of SCF (i.e. under non-differentiating conditions). Within 3–4 weeks, 39% of clones were dead or dying, 39% had differentiated and the remainder underwent extensive cell death before subclones emerged, either looking and growing like the parental EML cells or else differentiating. Figure 3E shows growth histories for 37 clones still alive after 40 days. Each point reflects the state of the culture on the indicated day post-cloning: “EML-like” cells grew rapidly in suspension (<5% adherent, blue circles), “mixed” cultures contained less than 50% differentiated (adherent) cells (purple circles), “differentiated” cultures contained mostly adherent cells (red circles) and “dying” cultures contained a majority of dead cells (black circles). Clones with lower levels of *Crebbp* message (as measured by qRT at week 5) were more likely to differentiate and eventually die. Clones that survived for the full length of the experiment (120 days) generally grew in suspension like parental EML cells and seemed to arise as subclones in cultures that had undergone substantial cell death. As controls, we also followed 20 clones receiving control shRNA targeting GFP and 20 that were cloned but not exposed to virus. All grew like parental EML cells for more than a month, indicating that differentiation and death were not predominantly side-effects of lentiviral treatment or cloning.

Down-regulation of CREBBP in EML cells thus mimics the mix of cell death and myeloid differentiation seen in these cells when they are driven to differentiate by ATRA, IL-3 and GM-CSF stimulation[53], suggesting that CREBBP either acts downstream of the growth factor signaling cascade or can trigger differentiation through an independent, parallel pathway. The spontaneous myeloid differentiation of EML cells after *Crebbp* knock-down, reminiscent of the increased myelopoiesis seen in *Crebbp*+/**−** animals[3], and the differentiation-induced down-regulation of CREBBP protein levels, similar to that observed in differentiating hematopoietic cells (Figure 3A and [57]), both indicate that regulated reduction of CREBBP abundance is a key event in early hematopoietic differentiation.

### Fluctuations in intronic-to-total mRNA ratios and protein levels in HSC-like EML cells depending on differentiation stage and CREBBP levels

We had confirmed by qRT that intron levels for *Itga4*, *Msi2* and *Tcf4*, each of which has been implicated in hematopoietic development[38], [41], [58], [59], were reduced in *Crebbp*+/**−** FL HSCs relative to WT (Figure 2). We now wanted to know whether a natural reduction in CREBBP protein occurring as a consequence of normal differentiation processes in EML cells would result in similar intronic changes. More importantly, we wondered whether fluctuations in intronic levels could have a measurable impact on protein levels.

**Figure 4:**
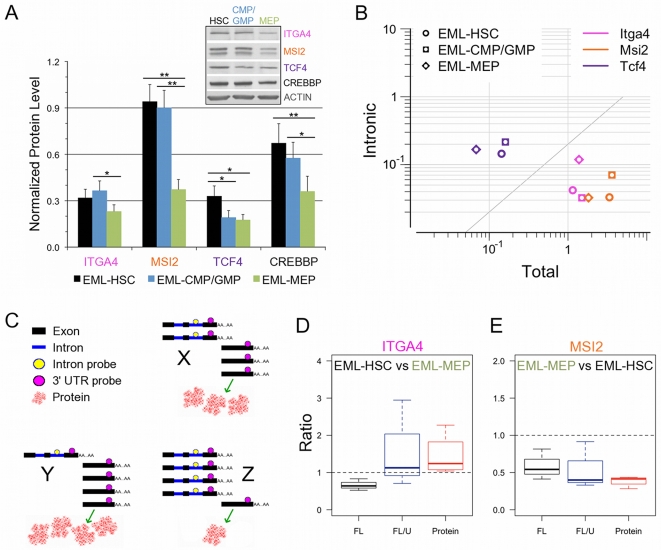
Regulated changes in protein levels and intronic sequence abundance as a function of differentiation stage. (A) Quantification of expression levels for the indicated proteins in phenotypically HSC-like cells (EML-HSC: Lin- SCA1+ CD34+ KIT++), early myeloid progenitors (EML-CMP/GMP: Lin- SCA1- CD34+ KIT++) and megakaryocyte/erythrocyte progenitors (EML-MEP: Lin- SCA1- CD34- KIT++). Bars indicate means + SEM of 4 independent experiments normalized to beta-actin levels. Significant differences in protein levels between cell types indicated by * for ANOVA p-values <0.05 and ** for <0.01. Inset: representative Western blots. (B) Full-length *vs* intronic qRT signals for the indicated genes in the EML subpopulations described in A. Shown are median values from 3 independent experiments. (C) Model of the potential impact of varying intronic levels for a constant level of full-length mRNA. Exonic sequences are represented by black rectangles and introns by blue lines. Probes for intronic and full-length sequences are indicated by yellow and magenta circles, respectively. For each of X, Y and Z, the full-length level is the same (5 magenta circles) but the proportion of unspliced transcript varies (X = 2 probes, Y = 1 and Z = 4). The corresponding protein levels (pink blobs) reflect the relative proportion of spliced and unspliced transcript since only the latter can be translated. (D) Ratio of full-length mRNA (black box, F), full-length/unspliced (blue box, F/U) and protein (red box, P) in EML-HSC relative to EML-MEP for *Itga4* (E) As in (D) but for *Msi2* in EML-MEP *vs* EML-HSC. Whisker plots in (D) and (E) show median (line inside box), interquartile range (limits of the box) and data range (limits of whiskers).

EML cell cultures kept undifferentiated by the presence of SCF are a mixture of CD34+, SCF-responsive, replicating cells and their progeny CD34-, SCF-independent cells that become responsive to interleukin-3[53]. In addition to HSC-like cells (EML-HSC: Lin- SCA1+ CD34+ KIT++), the cultures also contain cells which have cell surface marker profiles similar to early myeloid progenitors (EML-CMP/GMP: Lin- SCA1- CD34+ KIT++) and megakaryocytic-erythroid progenitors (EML-MEP: Lin- SCA1- CD34- KIT++). Down-regulation of CREBBP abundance can be seen in phenotypically distinct subpopulations of EML cells (Figure 4A, inset). In particular, EML-MEP have roughly 50% of EML-HSC CREBBP protein levels, much like primary BM MEPs do relative to HSCs (Figure 3A). In each case, protein levels were lowest in EML-MEPs (Figure 4A). Relative mRNA and intronic signals varied by target and cell subpopulation but, again, changes were most pronounced in EML-MEPs (Figure 4B).

Our model, based on the above results, is that the regulated production of unspliced, full-length transcripts can represent a sufficiently large proportion of the total mRNA pool to have a detectable impact on protein levels. Figure 4C shows a cartoon of scenarios in which relative protein abundance and total mRNA levels would not correlate well. In each case illustrated (X, Y and Z), the same full-length mRNA signal is present (as detected by qRT, for example, by a 3′ exonic probe indicated by the magenta spots). However, varying levels of unspliced transcript detected by an intronic probe (yellow spots) would result in different protein levels since only fully processed mRNA can be translated. In the absence of some correction for the presence of unspliced transcripts, a comparison of full-length message levels in X, Y and Z would thus predict lower than observed protein levels for Y relative to X or, conversely, higher than observed protein levels in Z relative to X.

Our data show instances of each of these cases. Full-length transcript levels of *Itga4* in EML-HSCs are on average lower than in EML-MEPs but protein levels are higher (Figure 4D). Factoring in intronic signal levels yields a better estimate of protein abundance. In the case of *Msi2* (Figure 4E), the combination of both lower total mRNA and a greater proportion of unspliced product in EML-MEPs relative to EML-HSCs results in a more substantial reduction in protein levels than predicted solely by relative mRNA levels.

Taken together, these data indicate that the differences in unspliced pre-mRNA levels we detected originally in FL HSCs are context-sensitive, varying with both developmental and differentiation stage. More importantly, these changes have the potential to alter protein levels and, consequently, to have an impact on downstream HSC functions.

## Discussion

The production of mature, fully spliced mRNA is subject to regulation not only at the initiation stage but also during elongation, 3′-end processing and through splice site selection. Although there is a preferred order in intron removal, excision does not necessary proceed in a linear or directional manner[60] and, in some cases, splicing of one intron can alter subsequent splice site selection[61], [62]. In addition, elongation and splicing are regulated in a cell type- and differentiation stage-specific manner[63]. Recent studies in HSCs[24], macrophages[18] and T-cells[19] have raised the possibility of a further level of control to this already complex system whereby production of full-length but unprocessed transcripts can serve to maintain a locus accessible but non-productive until appropriate signals are received. The role of CREBBP as a context-dependent transcriptional coactivator or inhibitor has been known for some time[64], [65]. Less familiar are its function in 3′-end processing[16] and its colocalization with splicing factors and nascent transcripts in nuclear speckles[14], [66]. Hargreaves et al. have also recently proposed a role for CREBBP in macrophages in the production of unspliced mRNA in primary response genes with GC-rich promoters (so-called PRG-I genes) [18].

In light of these studies linking CREBBP not only with initiation of transcription but also with pre-mRNA processing, we set out to determine whether fluctuations in intronic signals we detected in microarray studies of *Crebbp*+/**−** FL HSCs were real and might reveal something novel about the underlying biology of HSCs beyond what corresponding mRNA levels could tell us. Although our initial observations came from fetal HSCs, we found that the same probe set signature could distinguish adult HSC from differentiated cell types better than non-intronic probes sets for the same genes. In addition, despite the fact that each independently published data set we examined had slightly different isolation procedures for their HSC populations, our intronic signature consistently separated them from progenitors and mature cells, indicating that it reflects functionality rather than a specific cell surface phenotype.

Reduction in CREBBP levels did not result in a generalized defect in splicing, however. In fact, for most of the transcripts we tested by qRT, the level of intronic signal tracked with changes in total mRNA levels so that the proportion of unspliced message remained constant regardless of expression level. Nevertheless, in both FL HSCs and phenotypically immature EML cell subpopulations, there was a subset of genes that deviated from this linear relationship. Our results in FL HSCs had suggested that reduced levels of CREBBP triggered increased splicing since altered intronic probe sets were invariably down-regulated but results with EML cells indicate that, like other mechanisms regulating pre-mRNA production and maturation, the process (or combination of processes) we are detecting varies by target gene, cell type and differentiation state.

A further indication that the changes in intron levels we found in FL HSCs are functionally relevant is the fact that the transcription factor binding sites enriched in the proximal promoter regions of intronic genes were in part different from those for genes in which total mRNA abundance changes. Binding sites for SOX17, a factor essential for fetal but not adult hematopoiesis[52], were over-represented only in intronic targets. Also selectively enriched in intronic target promoters were binding sites for interferon-responsive factor 1 (IRF1) and the glucocorticoid receptor (NR3C1). Interferon alpha has been shown to trigger HSC cycling[67] and IRF1 and NR3C1 exhibit synergistic or inhibitory crosstalk in signal transduction depending on cellular context[68], [69]. One further tantalizing connection is that mice haploinsufficient for *Crebbp* expression develop myelodysplastic disease within their first year of life (manuscript submitted) and IRF1 has also been implicated in the pathophysiology of immune-mediated marrow failure in myelodysplastic syndrome[70] and is frequently deleted in 5q- myelodysplasia[71].

Interestingly, binding sites for the Ets family factors SFPI1/PU.1, ETS1, GABPA and ELK1 were enriched in down-regulated intronic targets and in the up-regulated mRNA set but not in down-regulated mRNA targets. This shared regulation by up-regulated mRNAs and transcripts with reduced intronic levels would make sense if, as our data suggests, a reduction in intronic signal results in greater mRNA splicing leading to increased protein production. Shared promoter elements would thus allow coordinated expression by directly up-regulating transcription for some targets while increasing the processing of unspliced transcripts for others. This partitioning of transcription factor binding site clusters between intronic and mRNA targets is worthy of further study as it points to specific regulatory modules participating in CREBBP-mediated control of mRNA processing.

It is still unclear whether the link between altered intronic signal and differentiation is causative but what is clear from our data is that knocking down *Crebbp* expression pushes multipotent EML cells to commit to myelopoiesis. The HSC-like EML cell line was established by introducing a dominant-negative retinoic acid receptor (RARα403) into mouse BM. Due to an RARα403 block, EML cells require treatment with ATRA to restore their ability to produce granulocyte-macrophage progenitors (CFU-GM) in response to IL-3 and GM-CSF[26]. Targeted reduction of *Crebbp* expression levels, however, allowed EML cells to bypass this block and differentiate into macrophages even in the presence of SCF doses normally able to maintain the cells in an undifferentiated state. This spontaneous myeloid differentiation of EML cells after *Crebbp* knock-down is reminiscent of the increased myelopoiesis seen in *Crebbp*+/**−** animals[3] and the differentiation-induced down-regulation of CREBBP protein levels is similar to that we observed in differentiating hematopoietic cells.

We have shown here that both splicing changes and spontaneous myeloid differentiation are sensitive to CREBBP dosage and selective alterations in abundance of intronic sequences correlate with differentiation in EML cells and changes in protein levels. Splicing changes varied by cell type and gene but nevertheless appear regulated as opposed to being a surrogate measure of total mRNA. Our data are consistent with a bookmarking model of HSC lineage priming with CREBBP involved in multiple steps of the lineage decision-making process as coactivator of transcription and coregulator of pre-mRNA processing. They also fit well with the concept that key regulators such as CREBBP can alter the landscape of HSC fate decisions[25] by altering the probability of adopting a given path towards self-renewal, death or differentiation.

## Materials and Methods

### Animals

Mice were bred and maintained under pathogen-free conditions at the animal facility of the GCCRI. All animal procedures were approved by the University Health Science Center Institutional Animal Care and Use Committee (protocol numbers 06030 and 06059). *Crebbp+/* **−** and *Ep300+/* **−** mice (kindly provided by Dr. D. Livingston, Dana-Farber Cancer Institute, Boston, MA) are fully back-crossed on a C57BL/6 background. WT littermates served as controls. B6;129S2-*Cdkn1a^tm1Tyj^*/J mice and B6129SF2 WT controls were purchased from Jackson Laboratories (Bar Harbor, ME).

### Isolation of FL HSCs and MEFs

Mated females were checked daily for the presence of vaginal copulation plugs, which defined day 0.5 of gestation. Plugged females were sacrificed on day 14.5 of gestation and their embryos harvested. The average cell count of 21 WT fetal livers (14 litters) was 21.3±4.1×10^6^ cells, indicating that the embryos were at a similar stage of development when sacrificed[72].

#### FL HSCs

Single cell suspensions were prepared from each FL and viably frozen. Once enough embryos of each genotype were collected, cells were thawed and prepared for HSC isolation. Lineage negative (Lin-: Ter119-, CD5- or CD4-CD8-, B220-, GR1-) SCA1+ AA4.1+ KIT++[73] cells were purified by fluorescence-activated cell sorting on a FACSAria (BD Biosciences, San Jose, CA).

#### MEFs

Day 14.5 post-coitus embryo bodies were disaggregated in 0.25% trypsin and plated in MEF medium (DMEM, 15% FBS, 2mM glutamine, 100 µM non-essential amino acids, 8.9×10^−5^M β-mercaptoethanol, penicillin/streptomycin; Invitrogen, Carlsbad, CA).

### Microarray analysis

All data is MIAME-compliant and the arrays are available for download from the Gene Expression Omnibus (GSE27987). For each HSC sample, 12 ng of RNA were amplified using the Ovation RNA amplification kit (NuGen Technologies, Inc., San Carlos, CA). MEF RNA was isolated using TRIzol (Invitrogen) and used without amplification. After biotin labeling, samples were hybridized to Affymetrix Gene Chip Mouse Genome 430 2.0 microarrays. Each microarray was checked for large-scale defects, RNA degradation and the proportion of probe sets called “Present” or “Absent” using MAS5 (∼50% in all cases). All arrays were comparable and satisfactory by these metrics. Arrays for each group were normalized and corrected for background using the Bioconductor[74] *gcrma* package. We excluded from further consideration probe sets for which all samples in a group were called “Absent”, for which all samples had intensities below log2 (100) or for which the interquartile range was <0.5. In the case of *Crebbp*+/**−**, *Ep300*+/**−** and their WT control FL HSCs, the values of the technical replicates were averaged prior to filtering based on minimum intensity levels and variation. Paired Student T-tests with p-value <0.05 and a fold-change >1.5 were used as the cut-off for calling significant change.

### Annotation

Probe sets on the arrays used in these studies typically comprise 11 25-mers designed to interrogate GenBank mRNA and EST sequences. We used Blast[75] to map the individual 25-mers for each probe set to mouse RefSeq mRNA sequences (May 2009). Probe sets that did not match reference mRNA sequences were then aligned to genomic sequences corresponding to RefSeq genes (all bases between the 5′-most transcription start site and the 3′-most end position) based on Mouse genome Build 37 (Mm9, July 2007). In both cases, a probe set was called a match if at least 8/11 25-mers aligned perfectly.

### Hierarchical clustering of published data sets

Data sets of hematopoietic populations using the same microarray platform as our studies were downloaded from the Gene Expression Omnibus (http://www.ncbi.nlm.nih.gov/geo/) and either reprocessed from image files (GSE17765[30], GSE6506[31]) or the normalized data used as published (GSE18669[32]). Two sets of probe sets were used for clustering subpopulations: (a) intronic probe sets differentially expressed in *Crebbp*+/**−** vs WT HSCs and (b) the most variable mRNA probe set for each gene corresponding to an intronic probe set in (a). GenePattern[76] was used for hierarchical clustering and visualization. For randomization studies, the *hclust* and *cutree* functions from the R *stats* package[77] were used to generate dendrograms of Pearson correlations and determine the separation of HSCs from other cell types for 10,000 random samples of expressed intronic probe sets.

### Gene set overlap and TFBS analysis

Gene lists were submitted to web-based MSigDB[78] and DAVID[79], [80] applications and related or overlapping terms collapsed for simplicity of presentation. Vertebrate position-specific weight matrices were downloaded from JASPAR[81] and supplemented with Transfac[82] matrices for GFI1 and LEF1. These were used with Clover[83] to quantify over-representation of TFBS in target proximal promoters extending 500bp upstream and 250bp downstream of the transcription start site (TSS). We used two backgrounds to correct for compositional biases: one comprising 5Kb regions centered on all RefSeq (May 2008 download) and another consisting of mouse chromosome 9. Background sequences were derived from mouse NCBI Build 37 (Mm9). Results for intronic vs exonic targets were compared to identify TFBS uniquely enriched in intronic targets.

### EML cell culture and differentiation

EML cells (EML clone 1, CRL-11691) were obtained from ATCC (Manassas, VA). Cells were cultured in IMDM medium supplemented with 20% FBS (06952; StemCell Technologies, Vancouver, BC, Canada) and rmSCF (100ng/ml, R&D Systems, Minneapolis, MN) to maintain them in an undifferentiated state (non-differentiation medium, NDM). Myeloid differentiation was induced by a 3-step protocol adapted from a previous study[26]. Briefly, on day 1 cells were switched from NDM to IMDM plus 20% FBS with a lower dose of rmSCF (50 ng/ml), 25 ng/ml IL-3 (R&D Systems) and 10 µM all-trans retinoic acid (ATRA; R2625, Sigma, St Louis, MI). On day 4, the medium was replaced with fresh medium containing only GM-CSF (25 ng/ml) and 7 days later 10 µM ATRA was added.

### shRNA-mediated knock-down of CREBBP in EML cells

A lentivirus-encoded shRNA targeting the sequence 5′-CAAGCACTGGGAATTCTCT-3′ in mouse *Crebbp* (shCrebbp) was created by cloning oligonucleotides into the FSIPPW vector as previously described[84]. A control lentiviral shRNA targeting enhanced green fluorescent protein (shGFP, 5′-AAGAACGGCATCAAGGTGAACTT-3′) was generated similarly. Both were packaged as previously described[85]. Co-transfection of 293TD cells was performed using Lipofectamine 2000 according to manufacturer's instructions (Invitrogen). Undifferentiated, early passage EML cells were split one day prior to infection. Virus-containing supernatant supplemented with 8 µg/ml protamine was added to the cells for ∼16 hours after which the cells were placed in fresh NDM. A second round of infection was performed ∼8 hours later using a flow-through infection protocol[86]. Culture medium was replaced with fresh NDM supplemented with puromycin (3 µg/ml) the following day to select for transduced cells. Surviving EML cells were cloned in NDM methylcellulose plus puromycin (M3234, StemCell Technologies) for 8 days, after which individual clones were picked and expanded in liquid NDM.

### EML subpopulation isolation

Phenotypically primitive EML subpopulations[53] were purified by first removing B220+, CD19+ and Ter119+ cells by magnetic separation (Miltenyi, Auburn, CA) and then incubating with SCA1-biotin, followed by streptavidin-APC-Cy7 and CD34-FITC (BD Biosciences). No anti-KIT antibody was necessary since all B220- CD19- Ter119- cells were KIT++. 7-Aminoactinomycin D (BD Biosciences) was used to exclude dead cells. Purification was done on a FACSAria (BD Biosciences).

### Quantitative RT-PCR

Total RNA from cloned EML cells was isolated by TRIzol extraction and purified with the Qiagen RNeasy kit (Qiagen, Valencia, CA). Total RNA from all other samples was extracted using the Qiagen RNeasy (Plus) Micro Kit as directed by the manufacturer. RNA was then reverse transcribed using the High Capacity cDNA Reverse Transcription Kit (Applied Biosciences, Foster City, CA). FL HSC was amplified using the Ovation RNA amplification kit. PCR was performed using either the SYBR Green PCR kit (Qiagen) or the GoTaq qPCR Master Mix (Promega, Madison, WI) on a 7500 Real-Time PCR System (Applied Biosciences). Data were analyzed using the 2^−ΔΔCt^ relative quantification method[87] and normalized to *Gapdh*. Primers were designed using the Primer3 web interface[88] (http://frodo.wi.mit.edu/primer3/). See Table 2 for primer sequences.

### Western blots

Anti-CREBBP antibody AC26[89] was kindly provided by Dr D. Livingston and anti-EP300 antibody RW128 was purchased from Upstate Biotechnology, Lake Placid, NY. Antibodies against ITGA4 (Ab65984), MSI2 (Ab76148), TCF4 (Ab72586) and ACTB (Ab6276) were purchased from Abcam (Cambridge, MA). Visualization of proteins was done by staining with a goat anti-mouse or goat anti-rabbit HRP (Bio-Rad Laboratories, Hercules, CA), as appropriate, and Pierce ECL Western Blotting Substrate (Thermo Scientific, Rockford, IL) or Amersham ECL Plus (GE Healthcare, Piscataway, NJ).

#### Hematopoietic cells

Whole cell lysates from 15,000 cells of each phenotype were suspended in RIPA buffer containing protease inhibitors (Roche Diagnostics, Indianapolis, IN) and PMSF (Sigma-Aldrich, St. Louis, MO) then loaded in each well after heat denaturation (10 min @ 70°C).

#### EML cells

Whole cell lysates from 4–5×10^5^ cells suspended in RIPA buffer with protease inhibitors and PMSF or 12.5 µg of nuclear extracts were loaded per well, as indicated.

#### Image quantification and statistics

In all cases, ImageJ software (http://rsb.info.nih.gov/ij) was used to quantify band intensities. Significance levels for the differences between cell types were determined by correlated-samples one-way ANOVA and Tukey HSD test using the web-based calculator at VassarStats: Website for Statistical Computation (http://faculty.vassar.edu/lowry/anova1u.html).

## Supporting Information

Table S1 **Annotation of Differentially Expressed Probe Sets in** ***Crebbp*** **+/− vs WT HSCs.** Detailed Excel table of differentially expressed probe sets including EST support for intronic sequences and expression levels for mutant and WT HSCs.
(XLS)

Table S2 **Quantitative RT-PCR Validation of Microarray Results.** Detailed Excel table of locations of primer sequences relative to microarray probe sets and qRT validation results for exonic and intronic targets. For intronic probe sets, the table also includes the length of potential poly(A) RT priming sites and predicted presence of intronic poly(A) signals.
(XLS)
